# Valproate, thalidomide and ethyl alcohol alter the migration of HTR-8/SVneo cells

**DOI:** 10.1186/1477-7827-4-44

**Published:** 2006-08-21

**Authors:** Ujjwal K Rout

**Affiliations:** 1 Division of Pediatric Surgery Research Laboratories, Department of Surgery and the Center for Psychiatric Neurosciences, UMMC, Jackson, MS 39216, USA

## Abstract

**Background:**

Valproate, thalidomide and alcohol (ethanol) exposure during the first trimester of pregnancy is known to cause several developmental disorders. All these teratogens are known to pass the placental barrier and interfere directly with the normal development of the fetus. However, these teratogens also alter the formation and function of the placenta itself which may in turn affect the proper nourishment and development of the fetus. Optimum development of the placenta requires adequate invasion of trophoblast into the maternal uterine tissues. Changes in the migratory behavior of trophoblast by maternal exposure to these teratogens during placentogenesis may therefore alter the structure and function of the placenta.

**Methods:**

In the present study, the effects of sodium valproate, thalidomide and alcohol on the migration of human first trimester trophoblast cell line (HTR-8/SVneo) were examined *in vitro*. Cells were cultured in the wells of 48-well culture plates as mono or multilayers. Circular patches of cells were removed from the center of the wells by suction, and the migration of cells into the wound was studied using microscopy. Effects of low and high concentrations of valproate, thalidomide and alcohol were examined on the healing of wounds and on the migration rate of cells by determining the wound areas at 0, 3, 6, 12, 24 and 48 h. Effects of drugs and alcohol on the proliferation and the expression levels of integrin subunits beta1 and alpha5 in cells were examined.

**Results:**

The migration rates of trophoblast differed between wounds created in mono and multilayers of cells. Exposure to teratogens altered the migration of trophoblast into mono and multilayer wounds. The effects of valproate, thalidomide and alcohol on the proliferation of cells during the rapid migratory phase were mild. Drug exposure caused significant changes in the expression levels of beta1 and alpha5 integrin subunits.

**Conclusion:**

Results suggest that exposure to valproate, thalidomide or alcohol during the first trimester of pregnancy may change the ultrastructure of the placenta by altering the migration of trophoblast cells and this effect may be mediated by drug- or alcohol-induced changes in the expression levels of beta1 and alpha5 integrin subunits.

## Background

Epidemiological findings and studies with animal models reveal that impaired growth *in utero *and size at birth are critical determinants of the onset of various disease processes later in life [1]. Optimum intrauterine growth depends on several factors including proper nourishment of the developing fetus by maternal blood through the placenta [2]. Accordingly, proper development of the placenta plays a significant role in determining the health and well being of the offspring. Trophoblasts, the outer layer of cells in the blastocyst, not only facilitate its attachment with the uterine epithelium but play a significant role in the formation of the placenta by invading the uterine tissue and differentiating into several cell types with endocrine, vascular, immunological or transport functions [3]. Therefore, interference with trophoblast invasion may result in abnormal development of the placenta resulting in suboptimal nourishment to the fetus.

Drugs cross the placental barrier by ultrafiltration, diffusion, active transport or by special processes, such as pinocytosis, or through breaks in placental wall and access the developing fetus [4]. Valproate sodium (VPA), used in the treatment of epilepsy and bipolar disease, is lipophilic, is actively transported into the trophoblast, and crosses the placenta through passive diffusion and by interacting with placental carnitine transporter [5-8]. Valproate therapy during pregnancy causes a wide range of congenital and behavioral malformations in children [9]. Animal studies demonstrate histopathological changes in the extraembryonic and embryonic tissues, such as necrosis of cytotrophoblasts and suppressed proliferation of fetal capillaries, following exposure to valproic acid [10,11]. The immunomodulatory and anticancer drug thalidomide (THA) also passes through the placental barrier and causes several defects in children including neurobehavioral problems, ear and limb malformations [12]. Although use of this drug during pregnancy is prohibited in the United States, it remains a potential teratogen in many parts of the world [13-15]. Exposure to thalidomide increases mitotic activity of cytotrophoblast, forming irregular masses of cells with or without syncytiotrophoblast surrounding it, forming structures identical to embryoid bodies [16,17]. Alcohol (ethanol; ALC) diffuses across the placenta and affects the developmental program of the fetus at many levels resulting in fetal alcohol syndrome [18]. Exposure to alcohol during pregnancy increases the number and size of trophoblasts and dilates cisterns of rough endoplasmic reticulum. Alcohol during pregnancy causes hyperplasia of capillary basal lamina, hypertrophy of trophoblastic basal lamina, irregular vascularization and hyperemia at the basal zone and labyrinth of placenta [19,20]. All three teratogens change the proliferation and migration of various cell types *in vitro *and *in vivo *[21-23] and cause placental pathologies [10,11,16,24-26]. Thus it is possible that these teratogens may alter the number and the invasion of trophoblasts, changing placental ultrastructure.

To date, no study has been conducted to examine the effects of these teratogens on the migration and proliferation of human placental trophoblast. Therefore in this study, the effects of valproate, thalidomide and alcohol on the migration and proliferation of first trimester trophoblast cell line were examined *in vitro*. Because dose of toxicant is a critical determinant of developmental toxicity and is likely to be a key factor responsible for interspecies variability in response to many test agents [27], both low and high concentrations of drugs and alcohol were tested on the trophoblast cells. Since trophoblasts invade maternal tissues in multiple layers [28,29], migration assays were conducted both in mono and multilayers to examine the effects of drugs and alcohol on the migration of trophoblast in multiple layers. Moreover because changes in α_5_β_1 _integrin receptor-mediated adhesion are known to alter migration of trophoblast [30], the effects of valproate, thalidomide or alcohol exposure on the expression levels of β_1 _and α_5 _integrin subunits were examined in the trophoblast cell line in culture by Western blotting.

## Methods

### HTR cell culture and migration assays

HTR-8/SVneo cells were obtained from Dr. Charles H. Graham (Queen's University, Ontario, Canada). Cells were maintained in 75 cm^2 ^flasks (Fisher Scientific, Suwanee, GA) containing 40 ml RPMI 1640 medium supplemented with 10% fetal calf serum (both from Invitrogen, Carlsbad, CA), 200 μg/ml Streptomycin sulfate and 200 U/ml penicillin G sodium (Invitrogen) as described earlier [31,32]. Migration assays were conducted in wells of 48-well plates (Corning; Fisher Scientific) containing 500 μl culture medium. For mono and multilayer studies, approximately 600,000 and 1,800,000 cells respectively were plated in each well the day before the experiment. The next morning, cells in the center of the well were removed by suction using a sterile pipette tip. Unattached cells were removed immediately by removing existing medium in the well and washing the attached layer of cells twice with 500 μl of preincubated medium. Within 5 minutes, bright field images of the wells consisting of areas with removed cells were captured at 4× for zero hour data collection by a Nikon Eclipse Microscope supported by Metamorph software (Molecular Devices Corp., Sunnyvale, CA). Plates were incubated for 48 h in the absence (controls) or presence of valproate, thalidomide or ethyl alcohol and images of wells were captured at 3, 6, 12, 24 and 48 h of culture.

Stock solutions of Valproate (50 mg/ml) and thalidomide (56 mg/ml) were prepared in sterile water and DMSO respectively as suggested by the supplier (Sigma-Aldrich, St. Louis, MO). Further dilutions of valproate and thalidomide were conducted in the culture medium. Thalidomide at 100 μM contained 0.05% DMSO. Preliminary experiments were conducted to ensure that DMSO at this concentration did not alter the migration of cells in mono or multilayers within 48 h of culture (data not shown). Ethyl alcohol (200 proof; Sigma-Aldrich) solutions were prepared in the culture medium. High and low concentrations of these teratogens for the study were determined from published articles to ensure that the concentrations used would not increase cellular apoptosis and were suitable for migration assays [23,33-35]. At the completion of image acquisition, wound areas (μm^2^) not encroached upon by the migrating cells were derived using the drawing tool and algorithms of Metamorph software. Data were exported to Excel software and the change in area with time was represented as a percentage of zero hour data of each well. Migration rates (μm^2^/h) in control and treated trophoblast cells between two image acquisition times were determined by dividing the differences in percentage wound areas by the time (h) difference.

### RT-PCR detection of integrin subunit

Expression of various integrin subunits in the HTR-8/SVneo cells were examined by RT-PCR method. In brief, total RNA from monolayer of cells cultured for 12 h was isolated using RNeasy Mini kit (QIAGEN Inc., Valencia, CA). The concentration of RNA in solution was determined using a NanoDrop spectrophotometer (NanoDrop Technology, Inc., Rockland, DE). Total RNA (1 μg) was treated with DNase I to remove traces of DNA and subjected to reverse transcription using Superscipt III reverse transcriptase. Complementary DNA (cDNA) equivalent to 50 ng of total RNA was used for PCR reactions. Reagents used for cDNA synthesis and PCR reactions were from Invitrogen Inc. Primer sequences for the amplification of cDNA representing transcripts for α_2_, α_3_, α_4_, α_5_, α_6_, β_1 _and β_3 _integrin subunits were obtained from published articles [36-39]. *Primer3 *Input software () was used to determine sense (5'-GTGAGCTGCTTCAACATCCA-3') and antisense (5'-TCTCTCAAAGCCCTCGA CAT-3') primers for the amplification of α_IIb _integrin subunit mRNA from published human α_IIb _subunit cDNA sequence (Accession number M34480). Amplicon representing α_IIb _subunit mRNA from the HTR-8/SVneo cells were purified from the gel using QIAGEN gel extraction kit (QIAGEN, Inc.) and sequenced using a commercially available service (Retrogen, Inc., San Diego, CA). The sequence (genBank Accession number DQ841705) was subjected to BLAST search to confirm the identity.

### Cell proliferation assays

Because valproate, thalidomide and alcohol alter proliferation of cells in culture, effects of these drugs were examined on the number of HTR cells to determine influence of cell-number on the -migration data. Cells were plated in 96-well plates to examine the effects of valproate, thalidomide and alcohol on cell proliferation using a Cell-Quant kit (Invitrogen) and a VICTOR 1420 Multilabeled fluorescence detector (PerkinElmer, Fremont, CA). At 3 h, the first batches of assays were conducted to determine fluorescence intensities in wells plated with an increasing number of cells. This data was plotted to determine the linear range of the assay, and the slope was used to determine the relationship between the number of cells and fluorescence intensities. At 3 h, cells in some plates were treated with pre-equilibrated medium containing different concentrations of valproate, thalidomide, or alcohol. The medium of control wells was replaced with only pre-equilibrated medium. After 12 h, fluorescence intensities from untreated and treated cells were measured to determine the number of cells per well.

### Western blotting

Expression levels of integrin subunits were examined in cells at 3 h and 12 h after drug exposure. Cells were cultured in 6-well plates containing 5 ml of culture medium at 75% confluence. The next day, the medium was removed and replaced with 5 ml of pre-incubated medium supplemented with the drug or alcohol. Control wells were supplemented with medium only. High and low concentrations of each drug were used to determine the effects of concentration on integrin subunit expression. At the time of lysate preparation, incubation medium from control and treated wells was discarded. Attached cells were washed with cold phosphate buffered saline (PBS) and lysed with lysis buffer containing a proteinase inhibitor cocktail (Pierce, Rockland, IL). Lysate were centrifuged at 4°C and the supernatants were stored at -20°C. Protein concentrations in the supernatant were determined using a BCA protein assay kit (Pierce) on a NanoDrop spectrophotometer. Lysate supernatants were mixed with denaturing lane marker (Pierce) and heated in a boiling water bath for 5 min. Equivalent amounts of denatured proteins (10 μg) were subjected to 10% SDS-polyacrylamide gel electrophoresis and separated proteins were blotted onto nitrocellulose membranes using equipment and reagents from BioRad Labs. Membranes were blocked with 10% non-fat dry milk solution in PBS containing 0.1% Tween-20 (TTBS) and subjected to incubation with primary antibody (BD Biosciences Pharmingen, Franklin Lakes, NJ) in 5% blocking reagent overnight at 4°C. Membranes were washed three times with TTBS and exposed for 1 h to peroxidase conjugated secondary antibody (Jackson Immunoresearch, West Grove, PA). Membranes were washed in TTBS and treated with ECL solutions (Amersham Biosciences Corp., NJ) for the chemiluminescence detection of bands and for acquiring images in tagged format using a Kodak 440 imaging system. To avoid data fluctuations due to experiment-to-experiment variations in the intensity of bands from control and treated samples, membranes containing untreated and specific drug- or alcohol-treated samples were processed simultaneously for the detection of an integrin subunit. Membranes were first probed with the human reactive monoclonal antibody against β_1 _integrin subunit (clone 18) at 1:2,500 dilutions, stripped and reprobed with monoclonal antibody against α_5 _(clone 1) integrin subunit at 1:5000 dilutions. Both antibodies were obtained from BD Biosciences. Images were analyzed using ImageJ software  to determine the intensities of bands in arbitrary units. Intensity data representing the expression level of each integrin subunit from valproate-, thalidomide- or alcohol-treated cells were subjected to statistical analysis with the untreated samples of the same blot separately.

### Statistical analysis

Post-hoc test was performed using SPSS software (SPSS Inc., Chicago, IL) to compare individual mean ± standard deviations of mean values obtained by repeated measure ANOVA to determine the significance of difference. Differences between means at *p *< 0.05 were considered significant. Wound areas from 6 individual wells were obtained from 3 independent experiments each consisting of two controls and treated wells (N = 6). Cell numbers were determined from two independent experiments each consisting of 8 wells (N = 16). Band intensities for particular integrin subunit were determined from two independent experiments, each consisting of two replicates (N = 4).

## Results

### Migration of HTR-8/SVneo cells in mono vs. multiple layers (Figure 1)

**Figure 1:**
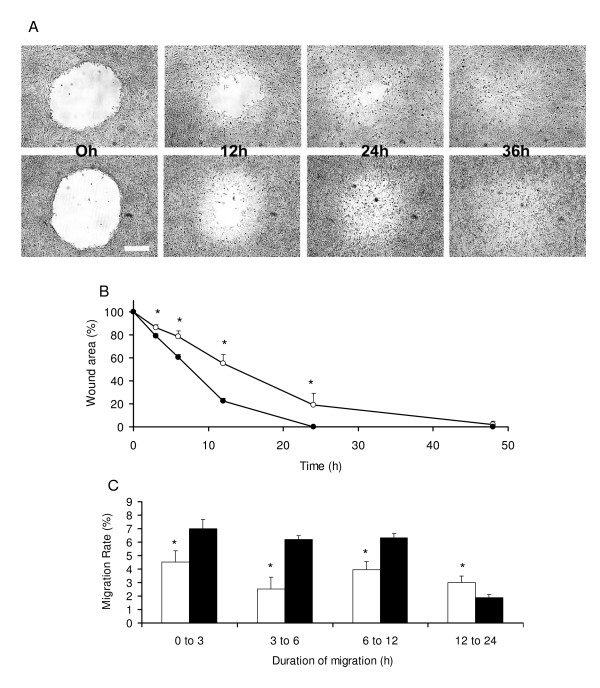
**Healing and migration rate of cells were faster in multilayer wounds**. **A**. Bright field images showing suction-wounds created in the monolayer (upper panel) and multilayer (lower panel) of cells at 0, 12, 24 and 36 h of incubation. Contrasts of images are increased to visualize the wound areas not invaded by the migrating cells. Bar Size 400 μm. **B**. Mean ± standard deviations of mean of wounds areas (μm^2^) during healing. Wound areas reduced to ~20% within 24 and 12 h of incubation in the monolayer (○) and multilayer (●) respectively. Complete healing of wound occurred earlier and by 24 h in multilayer cells. **C**. Mean ± standard deviations of mean of % migration rate of cells (μm^2^/h). Migration rate (%) in the multilayer wounds (■) remained higher until 12 h of incubation compared to those created in the mono layer (□) of cells. * *p *< 0.05

Suction wound areas (μm^2^) created in mono (1237279 ± 376135) and multilayers (934459 + 386014) of cells were not significantly different (*p *> 0.05). Wounds created in multilayers of cells healed faster than those in monolayer (Figure 1A). Wound areas were significantly lower in multilayer compared with monolayer cells as early as 3 h of creation and remained lower throughout the course, reaching about 1% at 24 h (Figure 1B). The earlier healing of wounds in multilayer cells was due to a significantly higher rate of migration (μm^2^/h) of cells, commencing as early as the initial 3 h of incubation (Figure 1C). The migration rate in the multilayer wounds remained higher until 12 h of incubation and then waned, healing the wounds slowly sometime during 24 to 48 h of incubation. Because most wounds in multilayer cells healed between 24 to 48 h of incubation and images were captured at 24 h and then at 48 h, it was not possible to calculate the exact time of complete healing and therefore to derive the rate of migration using the value of time (h). Due to this reason, the migration rates of cells in multiple layers during 24 to 48 h were not taken into consideration for the data analysis. Wounds created in the monolayer healed slowly but with a consistent rate except during 3 to 6 h of incubation when the migration rate of cells was lowest. Wounds in the monolayer were filled by 48 h. Percentage wound areas (%) at 3, 6 and 12 hours of incubation between the mono and multilayers of cells were significantly different (*p *< 0.05).

### Effects of valproate on HTR migration (Figure 2)

**Figure 2:**
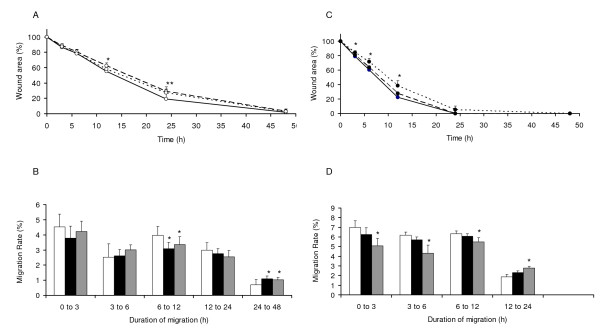
**Valproate reduced healing and migration rate of cells in mono (A & B) and multilayers (C & D)**. **A**. Mean ± standard deviations of wound areas (%) in monolayer cells. Wounds exposed to high concentration of valproate were significantly larger (*) than controls as early as 12 h of incubation and this difference was maximum by 24 h. Wounds exposed to low concentration of valproate were also larger than controls starting as early as 12 h of incubation but this difference was significant (**) only at 24 h of incubation. Differences in wound areas between the low and high valproate concentrations were not significant. **B**. Mean ± standard deviations of % migration rates (μm^2^/h) of cells in monolayer cells. Valproate at high and low concentrations changed the migration rate of cells throughout the course of assay. However, it was significantly reduced during 6 to 12 h and increased during 24 to 48 h. **C**. Mean ± standard deviations of wound areas (%) in multilayer cells. Wounds exposed to low concentration of valproate were significantly larger than controls at 3 h of treatments and this difference was at its maximum by 12 h of incubation. **D**. Mean ± standard deviations of % migration rates (μm^2^/h) of cells in multilayers. Both high and low concentrations of valproate inhibited migration rate of cells until 12 h. **Symbols **Monolayer wounds (○), Multilayer wounds (●), Control (-) or (□) High drug concentration (--) or (■), Low drug concentration (...) or (). *, ** *p *< 0.05.

Valproate inhibited healing in both mono (Figure 2A) and multilayers (Figure 2C) of cells, albeit more effectively in the multilayer wounds. In monolayer wounds, higher concentration (400 μM) of valproate inhibited migration more than at lower concentration (100 μM), whereas in multilayer wounds this effect was reversed. Further analysis of migration data of multilayer wounds revealed that this was due to a significant drop in the migration rate of cells (Figure 2D) treated with 100 μM VPA as early as 3 h of treatment. Later during 12 to 24 h, the migration rate of cells in multilayer wounds treated with 100 μM VPA increased but was not robust enough to overcome the delay. In monolayer wounds, both low and high concentrations of valproate inhibited the migration rate during 6 to 12 h of incubation (Figure 2B), but later (24 to 48 h) the migration rate of treated cells increased, filling the wounds almost at the same incubation time as controls.

### Effects of thalidomide on HTR migration (Figure 3)

**Figure 3:**
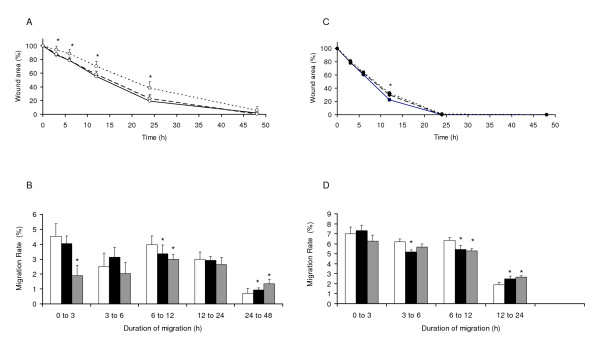
**Thalidomide reduced healing and migration rate of cells in mono (A & B) and multilayers (C & D)**. **A**. Mean ± standard deviations of wound areas (%) in monolayer cells. Wounds exposed to low concentration of thalidomide were significantly larger than controls by 3 h of incubation and this difference increased to maximum by 24 h of incubation. **B**. Mean ± standard deviations of % migration rates (μm^2^/h) of cells in monolayer. Migration rates of cells were significantly reduced by treatments with low concentration of thalidomide as early as 3 h of incubation. Later, during 6 to 12 h, the migration rate was reduced by low or high concentrations of thalidomide. At later phase of treatments (24 to 48 h), both high and low concentrations thalidomide increased the migration rate. **C**. Mean ± standard deviations of wound areas (%) in multilayer cells. Wounds exposed to low or higher concentration of thalidomide were significantly larger than controls (*) by 12 h of treatments. **D**. Mean ± standard deviations of % migration rates (μm^2^/h) of cells in multilayer. Migration rates during 3 to 6 h were significantly lower in wounds treated with high concentrations of thalidomide. Later, during 6 to 12 h, both low and high concentrations of thalidomide reduced the migration rate of cells. **Symbols **Monolayer wounds (○), Multilayer wounds (●), Control (-) or (□) High drug concentration (--) or (■), Low drug concentration (......) or (). * *p *< 0.05.

Thalidomide, like valproate, inhibited healing both in mono (Figure 3A) and multilayer wounds (Figure 3C), though more at lower (25 μM) than at higher (100 μM) concentration. This inhibitory effect of thalidomide on healing was more prominent with the monolayer wounds. Analysis of the rate of migration data revealed that both in mono (Figure 3B) and multilayer wounds (Figure 3D), thalidomide affected the migration rate significantly between 6 to 12 h of incubation at both low and high concentrations. However, the inhibitory effects of low concentrations of thalidomide commenced as early as 3 h of incubation in monolayer wounds. Nevertheless, in both control and treated cells, wounds healed almost at the same time, i.e., about 48 h in monolayer wounds and 24 h in the multilayer wounds.

### Effects of alcohol on HTR migration (Figure 4)

**Figure 4:**
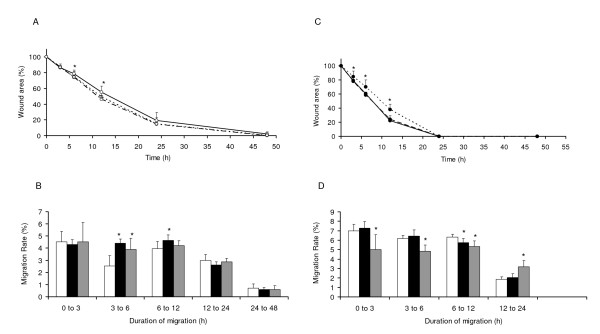
**Alcohol influenced healing and migration rate of cells differently in mono (A and B) and multilayers (C and D)**. **A**. Mean ± standard deviations of wound areas (%) in monolayer cells. Alcohol at high concentration significantly reduced wound areas in monolayer of cells by 6 and 12 h of incubation. Differences in areas between low and high alcohol concentration treated wounds were not significantly different. **B**. Mean ± standard deviations of % migration rates (μm^2^/h) of cells in monolayer. Alcohol at high and low concentrations increased migration rate of cells during 3 to 12 h of incubation. Later during 6 to 12 h of treatments, migration rates were increased significantly by only high concentrations of alcohol. **C**. Mean ± standard deviations of wound areas (%) in multilayer cells. Wounds treated with low concentration of alcohol were larger than the controls and those treated with high concentration of alcohol by 3 h of incubation and this difference was at its maximum by 12 h of incubation. **D**. Mean ± standard deviations of % migration rates (μm^2^/h) of cells in multilayers. In multilayer wounds, alcohol at low concentration inhibited migration rate of cells until 12 h. Later (12 to 24 h) the migration rate of cells treated with low concentrations of alcohol was higher than control and those treated with high concentration of alcohol. **Symbols **Monolayer wounds (○), Multilayer wounds (●), Control (-) or (□), High drug concentration (--) or (■), Low drug concentration (...) or (). * *p *< 0.05.

Effects of alcohol on healing was opposite between mono and multilayer wounds. Alcohol accelerated healing in monolayer wounds (Figure 4A), whereas it delayed healing in multilayer wounds (Figure 4C). The accelerating effects of alcohol on the monolayer wound healing were higher at high concentration (100 mM), whereas the inhibitory effects of alcohol on the multilayer wounds were only observed with low concentration of alcohol (25 mM). Analysis of the migration rate of cells revealed that the accelerating effects of alcohol on the migration rate of cells in monolayer wounds were most prominent between 3 to 6 h of incubation (Figure 4B). The inhibitory effects of low concentration (25 mM) alcohol on multilayer wounds were persistent until 12 h of incubation (Figure 4D). In both control and alcohol-treated cells, wounds healed almost at the same time, i.e., about 48 h in monolayer and 24 h in the multilayer cells.

Migration rate data (Figures 2B, 2D, 3B, 3D, 4B and 4D) presented in tabulated format (Table 1) revealed that the exposure of cells with a drug or alcohol influenced the rate of migration mostly during 6 to 12 h of incubation. Thalidomide consistently inhibited the migration rate of cells in both mono and multilayer wounds during this time period. Exposure to low concentrations of valproate or alcohol invariably altered the migration rate of cells in multilayer wounds during the entire incubation period, specifically inhibiting the migration rate during 0 to 12 h of incubation.

**Table 1:** Effects of drug and alcohol on the migration rate of HTR-8/SVneo cells

**Drug/Alcohol**	**Cell Layer/s**	**Concentration**	**0–3 h**	**3–6 h**	**6–12 h**	**12–24 h**	**24–48 h**
VPA	Mono	High	--	--	**↓**	--	**↑**
		Low	--	--	**↓**	--	**↑**
	Multi	High	--	--	--	--	**nd**
		Low	**↓**	**↓**	**↓**	**↑**	**nd**

THA	Mono	High	--	--	**↓**	--	**↑**
		Low	**↓**	--	**↓**	--	**↑**
	Multi	High	--	**↓**	**↓**	**↑**	**nd**
		Low	**↑**	--	**↓**	**↑**	**nd**

ALC	Mono	High	--	**↑**	**↑**	--	--
		Low	--	**↑**	--	--	--
	Multi	High	--	--	**↓**	--	**nd**
		Low	**↓**	**↓**	**↓**	**↑**	**nd**

**Arrows **Significantly different from the respective control at *p *< 0.05. (Direction of arrows shows changes in the migration rate); **nd **Migration rate not determined.

(---) Not Significantly different from the respective control.

### VPA, THA and ALC effects on the expression of integrin subunits in HTR cells (Figures 5 and 6)

**Figure 5:**
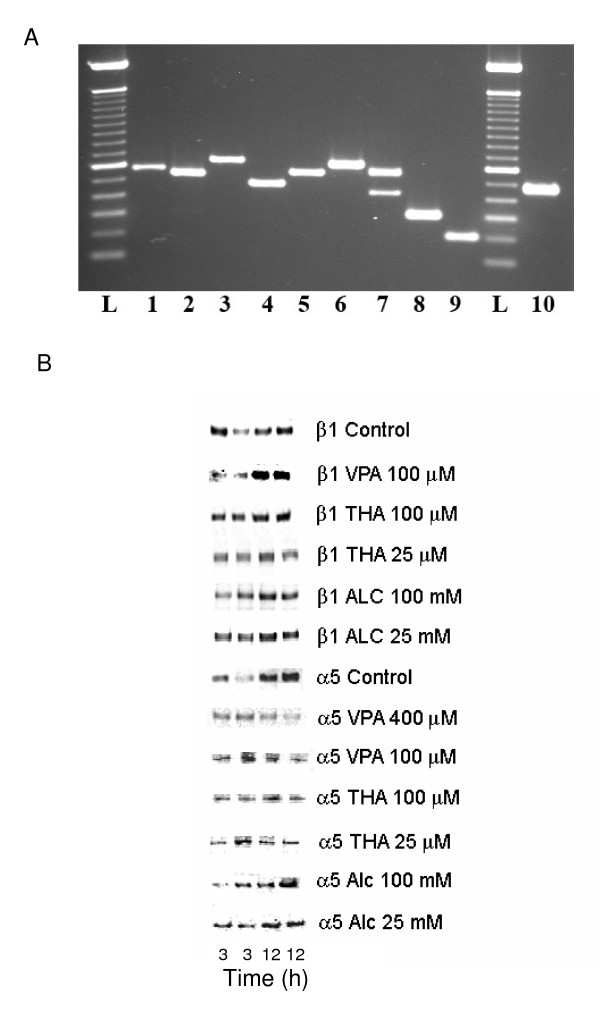
**Expression of integrin subunit mRNA species and drug effects on the expression levels of β_1 _and α_5 _integrin subunits**. **A. RT-PCR amplification of integrin subunit mRNA species**. Transcripts representing integrin subunits were amplified by RT-PCR. Amplicons subjected to Agarose gel electrophoresis, stained with ethidium bromide and photographed. Amplicons representing α_IIb _(570 bp), α_2 _(541 bp), α_3A_(656 bp), α_4 _(484 bp), α_5 _(564 bp), α_v _(619 bp), α_6 _(A 420 bp and B 550 bp), β_1 _(300 bp), β_3 _(200 bp) integrin subunits and β actin (478 bp) transcripts from HTR-8/SVneo cells are shown in lanes 1, 2, 3, 4, 5, 6, 7, 8, 9 and 10 respectively. L: 100 bp molecular weight markers. **B. Western blots of integrin subunits β_1 _and α_5 _in control and treated HTR cells**. Composite of representative blots showing expression levels of β_1 _and α_5 _integrin subunits in HTR cells at 3 and 12 h of incubation in absence (control) or presence of low or high concentrations of drugs or alcohol. Integrin subunit bands from two separate experiments (of total four), each consisting of lysate from 3 and 12 h incubation are shown in adjacent lanes. Treatment conditions and integrin subunits are mentioned on the right of each blot. Incubation periods of cells in absence or presence of drugs are shown below the composite.

**Figure 6:**
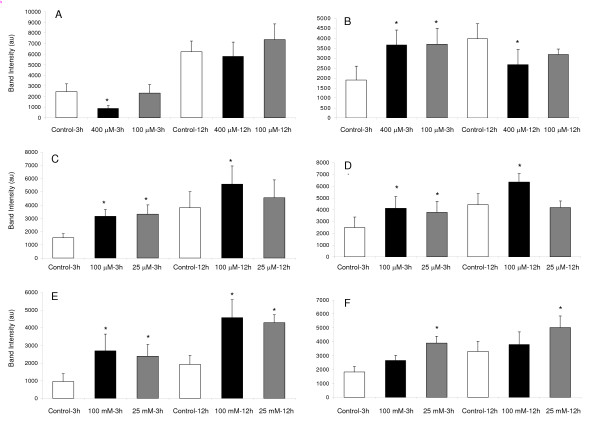
**Drug-induced changes in the expression levels of β_1 _and α_5 _integrin subunits**. Mean ± standard deviations of mean values of integrin subunit band intensities derived from 4 separate Western blotting experiments. Cells at 75% confluence were treated with low or high concentrations of valproate (A & B), thalidomide (C & D) or alcohol (E & F). Drug treatments in culture changed intensities of β_1 _(left panels) and α_5 _(right panels) integrin subunit bands. Drug concentrations and the time of exposures are shown below each bar. **Symbols **Controls (□), High (■) and Low drug () concentration. * *p *< 0.05.

Transcripts of α_IIb_, α_2_, α_3_, α_4_, α_5_, α_v_, α_6_, β_1 _and β_3 _integrin subunits were detected by RT-PCR experiments (Figure 5A). Bands representing integrin subunit β_1 _(~130 kDa) and α_5 _(~150 kDa) were detected in control and treated cells at 3 and 12 h of incubation by Western blotting (Figure 5B).

Expression levels of β_1 _(Figure 6; left panels) and α_5 _(Figure 6; right panels) integrin subunits increased with time in untreated cells (*p *< 0.05). Treatment of cells with valproate, thalidomide or alcohol changed the expression levels of β_1 _and α_5 _subunits, but the patterns of these changes were similar for thalidomide and alcohol treatments only. Exposure to high concentration of valproate for 3 h decreased expression levels of β_1 _subunit (Figure 6A) but increased expression of α_5 _subunit (Figure 6B). Treatments with high or low concentrations of valproate for 12 h did not alter expression levels of β_1 _integrin subunits, but decreased the expression levels of α_5 _subunit, albeit significantly (p < 0.05) with only the high concentration of drug.

Thalidomide and alcohol treatments for 3 and 12 h changed the expression pattern of β_1 _and α_5 _subunits in a similar manner, though not always significantly. For instance, treatments with low or high concentrations of thalidomide for 3 h increased expression levels of both β_1 _(Figure 6C) and α_5 _(Figure 6D) integrin subunits. Furthermore, this pattern was maintained even when treatments were prolonged for 12 h, although the difference between the low concentration of thalidomide and untreated cells was not statistically significant (p > 0.05). Similar to thalidomide, exposure of cells to alcohol for 3 and 12 h increased expression levels of β_1 _(Figure 6E) and α_5 _subunits (Figure 6F), except that the increase in α_5 _expression with high concentration of alcohol treatment was mild and not statistically significant from the respective control (untreated cells). Therefore, the patterns of β_1 _and α_5 _expression levels in cells were similar following thalidomide and alcohol treatments for 3 and 12 h, but were different than those with valproate treatments. This is obvious from the Western blot data presented in the tabulated format (Table 2).

**Table 2:** Drug or alcohol induced changes in the expression levels of β_1 _and α_5 _integrin subunits in HTR-8/SVneo cells

**Drug/Alcohol**	**Treatment Period**	**Concentration**	**Integrin Subunit β_1_**	**Integrin Subunit α_5_**
VPA	3 h	High	↓*	↑*
		Low	--	↑*
	12 h	High	--	↓*
		Low	**↑**	**↓**
THA	3 h	High	↑*	↑*
		Low	↑*	↑*
	12 h	High	↑*	↑*
		Low	**↑**	--

ALC	3 h	High	↑*	**↑**
		Low	↑*	↑*
	12 h	High	↑*	**↑**
		Low	↑*	↑*

**Arrows **Expression levels of integrin subunit relative to respective control (untreated cells).

* Significantly different from the respective control at *p *< 0.05.

(---) No change from the respective controls.

### Effects of VPA, THA and ALC on the proliferation of cells (Figure 7)

**Figure 7:**
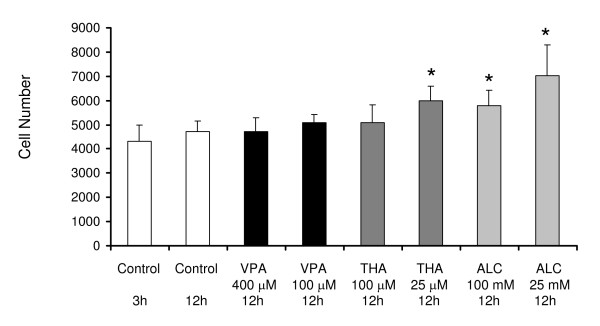
**Effects of Valproate, Thalidomide and Alcohol on the proliferation of trophoblast**. Mean ± standard deviations of the number of HTR cells in culture in the absence and presence of a drug or alcohol. Number of cells between 3 and 12 h of incubations were not significantly different (*p *> 0.05) in untreated cells (controls). Treatments with low and high concentrations of valproate for 12 h did not alter cell numbers from untreated cells (12 h control) significantly (*p *> 0.05). Treatments with thalidomide at low concentration and alcohol at both low and high concentrations for 12 h increased number of cells significantly from the controls. Treatment conditions and time of incubations are shown below each bar. * Significantly different from controls at *p *< 0.05. **Symbols **Controls (□), Valproate (■), Thalidomide (), Alcohol ().

Differences in the number of untreated cells between 3 to 12 h of culture were not statistically significant (*p *> 0.05). However, the number of cells treated for 12 h with low concentrations of thalidomide (25 μM) or low (25 mM) or high (100 mM) concentrations of alcohol increased significantly (p < 0.05) from the untreated cells cultured simultaneously for 12 h.

## Discussion

This study reports a novel way of studying cell migration *in vitro *using suction-wounds that can be created using a sterile tip and a vacuum unit. This method is superior to conventionally used scratch assays because wounds with comparable perimeter can be created in wells that allow examining cell migration and healing in relatively similar wounds. In addition, the entire wound area can be visualized under low magnification (4×) for study. This obviates measurement errors during imaging at different times of culture that is common with conventional scratch assays and occur because of differences in the width of the wound along the length of the scratch.

The results suggest that valproate, thalidomide and alcohol may influence migration of human first trimester trophoblast. Therefore, exposure to these teratogens during the first trimester of pregnancy may interfere with the normal development of placenta. This may cause suboptimal nourishment of developing embryos resulting in developmental defects. Results demonstrate that the changes in the migration rate of human first trimester trophoblast after drug and alcohol treatments may result from the alteration in the expression levels of β_1 _and α_5 _integrin subunits.

Data presented here reveal for the first time that the migration rates of cells in monolayer, and as reported in several studies using scratch assays, differ from those of cells in multilayers, a situation that is relatively more realistic to what is seen *in vivo *[28,29]. Although experiments conducted do not explain the mechanisms for the differences in the migration rate of cells between mono or multilayer wounds, it is likely that the accelerated migration of cells in multilayers wounds is due to better enrichment of medium with the cell migration enhancing factors secreted by relatively larger number of cells per well. These molecules may include extracellular matrix proteins regulating trophoblast migration, cytokines (TGF-β), growth factors (IGF-II), decorin, plaminogen activators, endothelin-1 and hormones regulating trophoblast migration [40-48]. Therefore, migration of invading trophoblast *in vivo*, where they invade in multiple layers [28,29], may be favored by the sufficiency of these molecules.

All three teratogens tested in this study are reported to alter the migration of different cell types, albeit differently. *In vitro *tests show that valproate may increase or decrease the migration of different glioma cell lines [49,50] and neural crest cells individually or in sheets [49]. Thalidomide changes the migration of cells differently at different concentrations [51] and cell type. It increases migration of multiple myeloma cells and inhibits migration of human keratinocytes [52,53]. Valproate and thalidomide exposure during prenatal development causes abnormal positioning of serotonergic neurons in rats [22], and migration of cortical neurons in rat fetal brains are delayed following prenatal exposure to alcohol [21]. There are numerous reports on alcohol's ability to alter the migration of various cell types, including mouse trophoblast, in culture differently [23,54-58]. Therefore, it is obvious that these teratogens target cell migration machinery differently in different cell types depending upon the concentration and cellular milieu. Data presented here also demonstrate that drugs and alcohol change the migration rate of trophoblast differently depending upon concentration and cell density (Table 1). Therefore, it is possible that exposure to these teratogens during placentogenesis may influence invasion of first trimester trophoblast differently depending upon the dosage and the cellular surroundings. The disparity in the effects of low concentrations of alcohol on the migration of cells between mono and multilayers supports this possibility. Differences in the drug- or alcohol-induced changes in the migration rates between the mono and multilayer wounds may derive from the capacity of each drug to influence secretion of ECM and factors influencing migration machinery, and possibly may be due to their ability to change the cell-plate and cell-cell interactions.

Expression of integrin subunit α_6_, α_5 _and β_1 _mRNA, and the roles of α_6_β_1_, α_6_β_4_, α_1_β_1_, α_2_β_1_, α_5_β_1_, α_v_β_1 _and α_v_β_3 _integrin receptors regulating the migration of trophoblast were reported earlier [31,59-61]. Data presented here provide evidence for the expression of α_3A_, α_4_, and α_IIb _integrin subunit mRNA and splicing of α_6 _integrin transcripts in a first trimester human trophoblast cell line. Detection of α_IIb _mRNA in first trimester trophoblast cell line and a recent report describing the role of α_IIb_β_3 _integrin receptor in trophoblast migration in mice [28] implies possible involvement of this receptor in the migration of human trophoblast. Additionally, the existence of α_6A _and α_6B _mRNA splice variants in the human trophoblast cell line hint for additional regulatory controls of trophoblast invasion mediated by α_6_β_1 _or α_6_β_4 _receptors [61-63].

Western blotting data show that valproate, as well as thalidomide and alcohol, changes the expression levels of β_1 _and α_5 _integrin subunits. Therefore treatments with these drugs or alcohol may alter the migration of trophoblasts by changing the availability of subunits for the formation of active α_5_β_1 _integrin receptors on the cell surface. Western blot data presented in the tabulated format (Table 2) reveal that the pattern of changes in the expression levels of β_1 _and α_5 _subunits by thalidomide or alcohol are almost similar, whereas valproate altered the expression levels of β_1 _and α_5 _integrin subunits differently. Nonetheless, it is likely that exposure to these drugs and alcohol during first trimester of pregnancy may alter the invasion of trophoblasts by disturbing the subunit stoichiometry during the formation of functional α_5_β_1 _receptors.

Enhanced adhesion of trophoblasts by α_5_β_1 _receptor is shown to inhibit the invasion of human trophoblasts [30], but this model may not completely explain changes in the migration rate of trophoblasts following alcohol treatments. This is because alcohol increased α_5 _and β_1 _integrin subunit levels at both low and high concentrations but accelerated migration of trophoblasts in monolayer and inhibited in multilayer wounds. These results indicate that expression levels of β_1 _and α_5 _integrin subunits may not be the sole determining factor for the migration rate of cells. Obviously, changes in the expression pattern of other integrin subunits α_1_, α_2_, α_v_, α_6_, α_IIb_, β_3 _and β_4 _that are known to be expressed in trophoblast [28,30,62,63], and down-stream molecules regulating integrin-mediated migration may also account for these differences. Besides, because cell migration is a dynamic process, changes in the expression of these molecules may not be steady during the course of migration. Therefore, studying steady state levels of integrin subunit expression pattern may not completely explain the migratory behavior of cells. Studies of molecules regulating cell migration in real-time in untreated and treated cells may be required to further clarify the mechanisms.

Valproate, thalidomide and alcohol influence proliferation of various cells [53,64-68]. Therefore, changes in the number of trophoblasts caused by these teratogens during the migration assay may influence the healing rates of the wounds. To examine the extent of this possibility, efficacy of low and high concentrations of teratogens on the number of trophoblasts was tested in a time frame relevant to the early phase of healing (3 and 12 h), when the migration rates were rapid. Data obtained indicated that changes in cell numbers due to treatments were mild and significant only at low concentrations of thalidomide or both at low and high concentrations of alcohol. Therefore, treatment of cells with these drugs is not likely to influence the migration rate of trophoblasts robustly. Besides, changes in the number of cells did not correlate with changes in the migration rate of cells. For instance, 12 h of treatment with 25 μM thalidomide increased the cell numbers significantly, but the migration of cells at this concentration was inhibited. Similarly, alcohol at low concentration increased the number of cells more than those treated with high concentration for 12 h, while the migration rate of cells in monolayer wounds was accelerated more by high concentration of alcohol during 3 to 12 h. These observations suggest that changes in the migration rate of cells were not influenced significantly by differences in proliferation rates induced by treatments, at least during the initial 12 h of incubation.

It is possible that *in vivo *effects of drugs on the migration and proliferation of trophoblasts may not be the same as those observed in this cell line tested *in vitro*. For instance, valproate inhibits human sperm motility differently *in vivo *as compared to *in vitro *conditions [69]. Maternal genotype regulating metabolism of drugs and alcohol may also influence the outcome [47,70]. In addition, the direct action of teratogens tested on cells in culture may not be same as those occurring due to the drug or alcohol and their metabolites *in vivo*. Therefore, data obtained *in vitro *will require verification in control and drug-exposed human placentas of different genotypes.

One of the likely consequences of poor trophoblast migration and invasion is the development of preeclampsia. Increased risk of preeclampsia due to valproate therapy is reported [71], but no association between thalidomide exposure or maternal alcohol drinking with preeclampsia is known so far. Therefore, it is likely that disturbances in the development of placenta due to changes in the invasiveness of trophoblast may not be the sole determining factors for the risk of preeclampsia. Of the three teratogens tested in this study, only valproate may influence these additional factor/s.

## Conclusion

All teratogens tested in this study are known to change the development and function of the placenta in human and animal models (see Introduction). Results from this study suggest that these placental pathologies may partly be due to alterations in the migration rate of trophoblasts by drugs or alcohol exposure, possibly mediated by changes in the expression levels of α_5_β_1 _integrin receptors in the trophoblast. Because wounds finally heal even under treated conditions, it is anticipated that trophoblasts attain their destination despite prenatal exposure to valproate, thalidomide or alcohol. Therefore, it is the temporal changes in the interaction of trophoblasts with the external milieu resulting from its altered pace of invasion that possibly adds to the placental anomaly.

## Abbreviations

ALC: Alcohol, VPA: Sodium Valproate, THA: Thalidomide.

## Authors' contributions

UK Rout planned the project, conducted experiments, analyzed data and wrote the manuscript.
